# Unintended consequences of cigarette price changes for alcohol drinking behaviors across age groups: evidence from pooled cross sections

**DOI:** 10.1186/1747-597X-7-28

**Published:** 2012-07-11

**Authors:** Deborah L McLellan, Dominic Hodgkin, Pebbles Fagan, Sharon Reif, Constance M Horgan

**Affiliations:** 1 Institute for Behavioral Health, Heller School for Social Policy and Management, Brandeis University, 415 South Street, MS035, Waltham, MA, 02454-9110, USA; 2 Cancer Prevention and Control, University of Hawaii Cancer Center, 677 Ala Moana Blvd. Gold Bond Building, Suite 200, Honolulu, HI, 96813, USA

**Keywords:** Cigarette price, Tobacco policy, Smoking, Drinking behaviors, Age, Young adults, Older adults

## Abstract

**Background:**

Raising prices through taxation on tobacco and alcohol products is a common strategy to raise revenues and reduce consumption. However, taxation policies are product specific, focusing either on alcohol or tobacco products. Several studies document interactions between the price of cigarettes and general alcohol use and it is important to know whether increased cigarette prices are associated with varying alcohol drinking patterns among different population groups. To inform policymaking, this study investigates the association of state cigarette prices with smoking, and current, binge, and heavy drinking by age group.

**Methods:**

The 2001-2006 Behavioral Risk Factor Surveillance System surveys (n = 1,323,758) were pooled and analyzed using multiple regression equations to estimate changes in smoking and drinking pattern response to an increase in cigarette price, among adults aged 18 and older. For each outcome, a multiple linear probability model was estimated which incorporated terms interacting state cigarette price with age group. State and year fixed effects were included to control for potential unobserved state-level characteristics that might influence smoking and drinking.

**Results:**

Increases in state cigarette prices were associated with increases in current drinking among persons aged 65 and older, and binge and heavy drinking among persons aged 21-29. Reductions in smoking were found among persons aged 30-64, drinking among those aged 18-20, and binge drinking among those aged 65 and older.

**Conclusions:**

Increases in state cigarette prices may increase or decrease smoking and harmful drinking behaviors differentially by age. Adults aged 21-29 and 65 and older are more prone to increased drinking as a result of increased cigarette prices. Researchers, practitioners, advocates, and policymakers should work together to understand and prepare for these unintended consequences of tobacco taxation policy.

## Background

Alcohol and tobacco use lead to enormous human and economic costs in the U.S. In the past few decades overall rates of smoking and drinking have declined [1,2]. However, alcohol contributes to about 98,000 deaths annually [3], and smoking, 443,000 deaths [4]. Annual direct and indirect alcohol-related costs approach $185 billion, while nearly $158 billion in health-related costs are ascribed to smoking [5,6]. Raising prices through taxation on tobacco and alcohol products is a common strategy used internationally to raise revenues and reduce consumption, especially among youth [1,2,7-12]. Taxation policies are product specific and focus either on alcohol or tobacco. An emerging body of economic literature, however, documents the interactions between the price of tobacco and use of alcohol [13-18]. These “cross-price” influences reflect a change in the demand for a good (e.g. alcohol) in response to an increase in price of another good (e.g. cigarettes) [19]. It is not known if increased prices on cigarettes are associated with binge or heavy alcohol drinking patterns across different age groups. To inform policymaking, it is important to know whether and how strategies to reduce cigarette use, such as cigarette taxation, influence drinking behaviors, and whether these effects differ across age groups.

### Underlying mechanisms influencing use and co-use of alcohol and nicotine

Biological, genetic, behavioral, socio-demographic, and policy factors can provide insight into understanding the link between alcohol and tobacco use and co-use that may, in turn, influence how tobacco control policies influence drinking behavior. Studies suggest there are biological and shared genetic components in using and becoming dependent on alcohol and nicotine both separately and together [20-23]. Behavioral mechanisms also link alcohol and tobacco, such as level of impulsivity, self-medication for psychological issues such as anxiety and depression, and reinforcement effects that help to explain how smoking and drinking can be triggers for each other [24].

Socio-demographic factors such as age, gender, race/ethnicity, education, socio-economic status, marital status, and employment status are associated with differences in smoking and drinking [25,26]. For example, younger people tend to have higher rates of smoking and binge drinking than those aged 65 and older [25,26], men have higher rates of smoking and drinking than women [25,26], Native Hawaiian/Pacific Islanders report the highest smoking and binge and heavy drinking rates compared to other racial/ethnic groups and Asian Americans report the lowest [25,26]. Studies show that full-time college students have the highest rates of binge and heavy drinking and those with college degrees have lower rates of smoking than those with 9-11 years of education [25,26]. Persons who are below the federal poverty level are more likely to smoke and less likely to drink than those who are above the federal poverty level [25,27]. Those who are in a partnership, and specifically those who are married, are less likely to have problematic drinking patterns and are less likely to be smokers [28-30]. Persons who are employed or out of the workforce (e.g. homemakers, students, retired individuals) are less likely to smoke than those who are unemployed [31]. The employed have higher rates of current drinking, but lower rates of problematic drinking than those who are unemployed [26].

Socio-economic and governmental practices and policies influence use. As an example, tobacco and alcohol companies use similar advertising and marketing practices which have been found to increase consumption of tobacco and alcohol [32,33]. Governmental policies impacting the pricing, taxation, and sales of tobacco and alcohol products have been shown to influence consumption of both products, individually and together [34,35]. Generally, higher prices reduce consumption of each product individually. Policies can also have impacts across products. For instance, tobacco control policies have been found to have effects on drinking behaviors. A study reported that smoking bans in bars are associated with higher traffic fatalities as drunk drivers travel further to bars allowing smoking [36].

Economics provides an understanding of further mechanisms influencing smoking and drinking. According to the theory of consumer demand, an increase in the price of a good (e.g. cigarettes) would be predicted to lower consumption of that good [37]. However, demand theory also recognizes that relationships between the demands for products may exist, so that when prices increase on one product it can have different effects on the use of both products, depending on the relationship. For instance, if two products are close “substitutes,” a price increase in one (e.g. cigarettes) will make a consumer buy more of the substitute (e.g. alcohol), and the cross-price relationship will be positive [37]. Alternatively, goods may be “complements,” in which case, when the price of cigarettes rises, the demand for both cigarettes and alcohol subsides, revealing a negative cross-price relationship [37].

In policy-making, cross-price relationships of goods may lead to intended and unintended consequences. For instance, increases in the price of cigarettes through taxation may decrease consumption of cigarettes (an intended consequence) and decrease harmful alcohol consumption if the goods are complements, arguably a positive unintended consequence. However, if the goods are substitutes and harmful drinking patterns amplify, then a negative unintended consequence might occur after cigarette prices increase. As policymakers may focus on the reduction of cigarette and alcohol consumption by youth and young adults as a reason for raising excise taxes, whether these taxes are associated with cigarette and alcohol use by young, in comparison with older persons, may be of interest to them.

While some literature examines the influence of cigarette prices on the prevalence and consumption of alcohol, there is no consensus among researchers on whether drinking increases or decreases as a result of cigarette prices [13,14,16-18,38]. One study found that higher cigarette prices increase drinking among adults over the age of 18 [13]. Others found a similar response among adults aged 51 and over [38], and among teens [18]. In contrast, some studies found that increasing the price of cigarettes reduces drinking [14,17,39,40]. Dee found that teens reduce drinking in response to increases in cigarette price, although in models with added controls, the relationship was no longer statistically significant [14]. A study of those 14 and older in Australia reported reduced drinking in response to increased cigarette prices as did studies in the U.K. and Sweden using sales data [17,39,40].

Only one of these studies investigated the impact of increases in cigarette price on current and binge drinking [14]. No studies have examined the influence of increases in cigarette price on different drinking patterns across various age groups. This is a notable gap since drinking and smoking rates vary dramatically by age. For example, the prevalence rates of binge drinking among adults aged 18-20 are 30-40%, are as high as 50% among adults aged 21-29, and are 23-30% among adults aged 30-49 [41]. After age 50 binge drinking drops; 12-19% of adults aged 50-64 and 8% of adults over the age of 65 binge drink [41]. While smoking prevalence rates are 20% for adults aged 18-64, they drop by half to 9.5% for persons over age 65 [42].

Given public health goals of preventing and reducing binge and heavy drinking, and the sharp differences in drinking patterns by age, this study aimed to address the significant gap in the literature on the impact of increased state cigarette price on alcohol drinking behaviors across age groups, with a particular focus on younger age groups. Since most of the U.S. research suggests that increases in cigarettes prices are associated with reductions in smoking and increases in drinking among the young, the primary hypothesis of this research was that increases in state cigarette prices would produce stronger reductions in current smoking, and stronger increases in current, binge, and heavy drinking among young adults (those under 30 years of age), than among those aged 30-64 and 65 and older.

## Methods

### Data and sample

This study analyzed six years (2001-2006) of nationally representative data from the Behavioral Risk Factor Surveillance System (BRFSS) surveys. Full details of the BRFSS design are available elsewhere [43]. The price and policy variables described below were merged into the BRFSS yearly data and then data were pooled. The BRFSS data identify the state in which a respondent resides—critical information for this study about impacts of state-level variables.

Due to different stratification sampling approaches, the U.S. territories were dropped from the sample, as were observations from Maine, New Hampshire, and Rhode Island (n = 74,581) which did not report results for the beer price variable. This yielded a sample size of 1,623,615 independent observations. Listwise deletion was used to address missing data (n = 299,857) [44], leaving a final sample of 1,323,758 for the analysis of data on current and heavy drinking. For binge drinking, the sample included 1,050,573, observations as we dropped observations from 2006 due to a change in definition of binge drinking for women in that year. The sample included users and non-users of cigarettes and alcohol.

### Measures

#### Current smoking and current, binge, and heavy drinking

The four dichotomous outcome measures were current smoking; and current, binge, and heavy drinking. Participation in current smoking; and current, binge, and heavy drinking were coded as 1 for survey participants who responded that they participated in these behaviors and coded 0 otherwise. During 2001-2006 the BRFSS defined current smoking as having smoked in the last 30 days, and current drinking as having at least one alcoholic beverage in the past 30 days [45]. For men, heavy drinking was defined in the BRFSS as having on average more than two drinks per day, and for women, more than one per day in the last 30 days. In 2001-2005, binge drinking was defined as having five or more drinks on one occasion in the last 30 days.

#### Cigarette price

The main explanatory variable was cigarette price, a continuous variable reflecting weighted average price of cigarettes (including generic brands) in dollars by state and year [46]. To correct for changes in price levels over time due to inflation, we deflated cigarette prices for the years 2001-2006 by the national Consumer Price Index (CPI) in constant 1982-4 dollars, the standard base year of the CPI used in the literature [47,48]. Reflecting other literature, the price included the federal tax, but did not include state and local taxes [46,49]. This variable was linked to the BRFSS state variable for each respondent.

#### Age

The age variable consisted of categories of those aged 18-20, 21-29, 30-64, and 65 years and over, in order to observe hypothesized variation between younger age groups (those under the age of 30) and older age groups. Binge and heavy drinking rates are highest among younger age groups and drop dramatically after age 65; an overall flattening of prevalence rates occurs between ages 30-64. Thus, we focus on differentiating the age groups of those under 30. Also, increases in excise taxes are passed by policy makers to reduce the prevalence of smoking and drinking among young people [1,10].

#### Other covariates

Additional individual-level variables were used to adjust for potential influences on smoking and drinking behaviors: gender; race/ethnicity; educational level; and partner, employment, and poverty status. Gender was a dichotomous variable, with males being the reference group. Race/ethnicity was an eight-category variable with Non-Hispanic whites as the reference group. The educational level variable had four categories with college graduates and above as the reference category.

Since drinking and smoking may vary by partner, employment, and poverty status, as described above, we used them for adjustment purposes. We collapsed them into categories that would have meaning for both alcohol and tobacco use. Partners (i.e. married or in an unmarried couple) were coded as 1 and those who were not in a partnership (i.e. divorced, widowed, separated, never married) were coded as 0. Employment status included the categories unemployed, employed (reference group), or not in the labor force. Poverty status was a categorical variable that was calculated using U.S. Department of Health and Human Services (DHHS) guidelines which included household income, household size, and state of residence [50]. The BRFSS income variable was an ordinal categorical variable grouped into eight ranges of household income. As the BRFSS categories did not exactly parallel the DHHS poverty guidelines, the “poverty status” variable in this study had three categories of those who were 1) definitely not poor, 2) definitely poor, and 3) may be poor. This third category of “may be poor” included those respondents whose category of household income straddled the cutoff points for definitely not poor, or definitely poor. Those with incomes that are definitely above the poverty level (i.e. definitely not poor) were the reference group. Other variables of theoretical interest were not included, either because they were highly collinear with existing variables (e.g. occupation), or were not available in the BRFSS (e.g. addiction level).

#### State-level policy and economic covariates

Beer price data from the American Chamber of Commerce Researchers Association (ACCRA) reported the retail price of a six-pack of Heineken and included federal, state, and local taxes, but excluded sales taxes and deposits [51]. ACCRA’s decision to use Heineken as the beer brand for beer price may not be reflective of all drinkers’ brand choice, since it is a medium-priced beer. Yet, these data are widely used in the literature [52] and are one of the only comprehensive sources of beer price over time. As with the smoking price variable described above, the beer price was in constant 1982-4 dollars (=1.00) adjusted for inflation.

We developed an ordinal variable measuring smoking restrictions (range 0-12) for each state. Drawing on prior work [47,53], the variable was calculated by identifying for each state the strength of smoking restrictions within private worksites, governmental worksites, restaurants, and bars [47,54,55]. We rated each venue on the strength of its smoking restriction, coded from 0 (no restrictions) to 3 (smoking is banned). Then the values for each of the four venues were added up to arrive at the value for the state smoking restriction variable. Local area restrictions were not included, as the dataset did not publicly identify respondents’ residences within states.

To adjust for overall trends in state-level economic indicators, yearly state poverty rates were included as a continuous covariate. State median household income and state unemployment rate were considered as potential co-variates, but because of their high correlations with other variables, they were excluded from final analyses. The four state-level variables that had the strongest support in the literature and the data available for inclusion remained: state cigarette price, state beer price, the magnitude of state smoke-free laws, and state poverty rate.

#### Other variables

Year and state dummy variables were included as fixed effects to account for other unmeasured factors at the state level that might have been correlated with smoking and drinking. The survey year (dichotomous as either 2001, 2002, 2003, 2004, 2005, 2006, or not) and state of residence (one out of a possible 47 possible states and the District of Columbia) were included as dichotomous variables. Finally, we weighted all data using the BRFSS weights. These weights adjust for non-response and non-coverage for geographical strata, to adhere to sample estimates of those geographical areas, and to take into account state stratification by age, gender, and race/ethnicity. Full details of the BRFSS design and weighting are available elsewhere [43].

### Statistical analyses

To test study hypotheses and address clustering that may occur at the state level over time, we estimated a series of linear regression equations using state and year as fixed effects [56]. A concern when conducting state policy research is that there could be confounding from unobserved characteristics of the state that are correlated with both smoking and cigarette price (e.g. anti-smoking sentiment). The benefit of using state effects, e.g. fixed effects, is that they account for any such unmeasured state-level characteristics that remain constant over time and may influence outcomes.

Univariate and bivariate analyses were conducted to determine variable means and relationships between cigarette price, age and smoking, and similarly for the three drinking variables. For each of the four outcome variables we estimated a linear probability model (LPM), using interaction terms (which interacted each age group with mean state cigarette price) to test hypotheses. All models included the same variables. Although the outcomes were binary, for which logistic regression usually is used, our inclusion of interaction terms introduces challenges with interpretation in nonlinear models. LPM has been found to be an appropriate and useful method for interpreting interaction terms when estimating models for binary outcomes [13,14,56-59]. The coefficients from each of four regression models were then used to derive the predicted response, separately by age group, of each behavior to a $1 increase in cigarette price, and expressed as percentage point changes.

All statistical analyses were performed using SAS version 9.2 (SAS Institute, Cary, North Carolina), utilizing procedures that address the complex sampling design of the BRFSS and adjust standard errors using the Taylor linearization method for computing variances [60]. The LPM models were estimated using OLS for weighted survey data (SAS PROC SURVEYREG). SAS options specified that models predicted the probability of smoking and drinking rather than the probability of not smoking or not drinking. The study was deemed exempt by the Brandeis University Committee for the Protection of Human Subjects.

## Results

Table 1 provides weighted descriptions of the sample, including cigarette pack price, socio-demographics and state-level policy variables. The six-year mean of the main independent variable, state cigarette price per pack, adjusted for inflation, was $2.02 (range, $1.95-2.09). Of importance for this study, 4.32% of the sample was aged 18-20, 16.49% aged 21-29, 64.27% aged 30-64, and 14.92% aged 65 and older. The analytic sample is slightly older compared to U.S. Census Data from 2002 [61].

**Table 1:** **Sample descriptive statistics among adults aged 18+,BRFSS, 2001-2006**^**(a)**^

**Variable**	**% or mean (SE)**
N = 1,323,758	
**Cigarette price, $ per pack**^**(b)**^, **by year and 6 year means**	
2001	$1.95 (0.00)
2002	2.09 (0.00)
2003	2.06 (0.00)
2004	2.01 (0.00)
2005	2.03 (0.00)
2006	1.98 (0.00)
6-year mean	$2.02 (0.00)
**Female gender, %**	50.54
**Age group,%**	
18-20	4.32
21-29	16.49
30-64	64.27
65 and higher	14.92
**Poverty, %**	
Not poor	84.89
Poor	8.91
Maybe poor^(c)^	6.19
**Race/ethnicity, %**	
Non-Hispanic White	71.14
Non-Hispanic African American	9.62
Non-Hispanic Asian	2.63
Non-Hispanic Native Hawaiian Pacific Islander	0.36
Non-Hispanic American Indian Alaskan Native	1.08
Non-Hispanic other race ^(d)^	0.78
Non-Hispanic multi-race	1.51
Hispanic	12.88
**Have partner, %**	64.10
**Employment status, %**	
Unemployed	5.05
Employed	64.64
Out of workforce	30.35
**Educational level, %**	
<High school degree	11.14
High school grad	29.45
Some college	27.28
College graduate or more	32.12
**State-level co-variates**	
Beer price, $ per six-pack ^(b)^	$3.99 (0.00)
Magnitude of state smoke-free laws (range 0-12), mean	4.17 (0.00)
State poverty rate, mean	0.12 (0.00)

Notes: BRFSS = Behavioral Risk Factor Surveillance System Surveys.

SE = Standard error.

(a) Weighted data are presented to reflect the complex sampling design of Behavioral Risk Factor Surveillance System (BRFSS).

(b) In 1982-4 dollars, adjusted for inflation.

(c) BRFSS income response categories do not track exactly with poverty guidelines limits, responses that were impossible to discern whether in poverty or not were coded as “maybe poor”.

(d) “Non-Hispanic other race” is not further defined in the BRFSS code book.

Table 2 shows how the weighted prevalences of smoking and drinking behaviors vary by age group. Overall smoking prevalence was 21.79%, drinking 55.38%, binge drinking 15.69%, and heavy drinking 5.43%. These overall prevalence rates mask statistically significant (p < .001) differences by age group for each outcome. Those aged 65 and older have lower prevalence rates of smoking, and current, binge, and heavy drinking than those younger than 65 years of age. Those aged 18-20 have higher prevalence rates of all four behaviors than adults aged 65 and older. Additionally, adults aged 21-29 have the highest prevalence rates of smoking and all three drinking patterns.

**Table 2:** **Smoking and drinking prevalence rates by age group, BRFSS, 2001-6**^**(a)**^

**Variable**	**Current smoking**	**SE**	**Current drinking**	**SE**	**Binge drinking**^(c)^	**SE**	**Heavy drinking**	**SE**
N = 1,323,758 ^(b)^								
**Total**	**21.79**	**0.00**	**55.38**	**0.00**	**15.69**	**0.00**	**5.43**	**0.00**
**Age group**^(d)^***								
18-20	24.19	0.49	47.05	0.58	25.26	0.55	8.40	0.35
21-29	27.58	0.22	63.95	0.24	28.99	0.25	8.44	0.15
30-64	22.92	0.08	56.99	0.10	14.48	0.08	4.97	0.04
65 and older	9.84	0.11	41.40	0.18	3.45	0.08	3.23	0.07

Notes: BRFSS = Behavioral Risk Factor Surveillance System Surveys.

SE = standard error.

(a) Weighted unadjusted percents are presented to reflect the complex sampling design of Behavioral Risk Factor Surveillance System (BRFSS) 2001-2006.

(b) N = 1,323,758. This is the sample used for all analyses, except as noted.

(c) N = 1,050,573. Includes observations for 2001-2005 only due to change in definition of binge drinking in 2006.

(d) Data represent chi-square statistics.

*** Age group has a statistically significant association with all four column variables (Overall chi-square df = 3, p < .001, for each column variable).

Regression results in Table 3 suggest that age group influences the associations between state cigarette price, smoking, and patterns of drinking. The cigarette price coefficient from the LPM results predicts the change in the probability of current smoking; and current, binge, and heavy drinking among the reference group (those aged 65 and older) from a one-dollar increase in the mean state cigarette price [56]. The coefficients on the interaction terms capture the differences from the reference group in the response of smoking and drinking behaviors to the state cigarette price. For example, the coefficient on “cigarette price x aged 21-29” (the interaction term) for binge drinking is 0.060. This indicates that the response of binge drinking to a one dollar increase in cigarette price is 6.00 percentage points higher for those aged 21-29 than it is for those aged 65 and older. Furthermore, the positive coefficients in Table 3 indicate that an increase in state cigarette price is associated with increases in the probability of smoking/drinking, while negative coefficients indicate associations with decreases in the probability of those behaviors. For example, for those aged 30-64, the negative coefficients on the interaction terms indicate that an increase in state cigarette price is associated with a reduction in the probability of current smoking and current drinking, compared to the reference group. However, the positive coefficient indicates that an increase in state cigarette price is associated with an increase in binge drinking among this population, compared to the reference group. Nearly all the coefficients on the interaction terms (e.g. cigarette price x aged 18-20) are statistically significant, indicating differences (both increases and decreases) from the reference age group (those 65 and older) in the response of smoking and drinking patterns to the state price of cigarettes.

**Table 3:** **Parameter estimates: smoking and drinking response to cigarette price by age group, BRFSS, 2001-6**^**(a)**^

**Independent variables**	**Current Smoking β (SE)**	**Current Drinking β (SE)**	**Binge Drinking**^**(c)**^**β (SE)**	**Heavy Drinking β (SE)**
N=1,323,758^(b)^				
**State cigarette pack price ($)**	0.014* (0.007)	0.037*** (0.009)	-0.019** (0.007)	0.001 (0.004)
**Age (reference = 65 and older**				
18-20	0.128*** (0.029)	0.215*** (0.034)	0.060 (0.032)	0.027 (0.019)
21-29	0.195*** (0.014)	0.264*** (0.016)	0.104*** (0.014)	0.027** (0.009)
30-64	0.211*** (0.008)	0.140*** (0.011)	0.054*** (0.006)	0.023*** (0.004)
Cigarette price x aged 18-20	-0.032* (0.014)	-0.077*** (0.017)	0.049** (0.016)	0.005 (0.010)
Cigarette price x aged 21-29	-0.006 (0.007)	-0.045*** (0.008)	0.060*** (0.007)	0.011* (0.004)
Cigarette price x aged 30-64	-0.025*** (0.004)	-0.029*** (0.005)	0.019*** (0.003)	-0.002 (0.002)
**Year (reference = 2006)**^**(d)**^				
2001	0.025*** (0.003)	0.022***(0.004)	0.002 (0.003)	0.002 (0.002)
2002	0.022***(0.003)	0.030***(0.004)	0.013***(0.003)	0.007***(0.002)
2003	0.021***(0.003)	0.043***(0.003)	0.016***(0.002)	0.007*** (0.002)
2004	0.008**(0.003)	0.028***(0.003)	0.005*(0.002)	0.002 (0.002)
2005	0.006**(0.002)	0.015***(0.003)	0	0.002 (0.001)

Notes: Models included covariates for gender, poverty status, race/ethnicity, co-habitating partner status, employment status, educational level, beer price (six-pack), magnitude of state smokefree laws, state poverty rate.

β = beta for parameter estimate; SE = Standard Error; *p < .05, **p < .01, ***p < .001, using t-tests, df =1,320,358 for current smoking, current drinking, and heavy drinking, df =1,047,879 for binge drinking.

(a) Weighted data are presented to reflect the complex sampling design of Behavioral Risk Factor Surveillance System (BRFSS).

(b) N = 1,323,758. This is sample used for all analyses, except as noted.

(c) N = 1,050,573. Dropped all observations from 2006 in binge drinking analyses to reflect change in definition of binge drinking for women in that year.

(d) Reference year is 2006 for all analyses except binge drinking analysis, when reference year is 2005.

Table 4 presents predicted responses by age group to a $1 increase in state cigarette price, based on the models estimated in Table 3. The results are expressed as predicted percentage point changes [56]. An increase in cigarette price was not associated with a significant change in smoking for those aged 18-29. However, a $1 increase in cigarette price was associated with a significant decrease in smoking of 1.10 percentage points among those aged 30-64, and an increase in smoking of 1.43 percentage points among those aged 65 and older.

**Table 4:** **Predicted smoking and drinking response to cigarette price—by age group, BRFSS 2001-6**^**(a)**^

	**Current Smoking**	**Current Drinking**	**Binge Drinking**	**Heavy Drinking**
	**Percentage point change**^**(c)**^	**Percentage point change**^**(c)**^	**Percentage point change**^**(c,d)**^	**Percentage point change**^**(c)**^
N = 1,323,758^(b)^				
**Age group**				
18-20	-1.76	-4.02*	2.98	0.56
21-29	0.83	-0.83	4.06***	1.15**
30-64	-1.10**	0.82	-0.09	-0.19
65 and older	1.43*	3.72***	-1.95**	0.05

Notes: Models included covariates for gender, poverty status, race/ethnicity, co-habitating partner status, employment status, educational level, beer price (six-pack), magnitude of state smokefree laws, state poverty rate.

BRFSS = Behavioral Risk Factor Surveillance System Surveys.

*p < .05, **p ≤ .01, ***p < .001, using t-tests, df =1,320,358, for current smoking, current drinking, and heavy drinking, df =1,047,879 for binge drinking.

(a) Weighted data are presented to reflect the complex sampling design of Behavioral Risk Factor Surveillance System (BRFSS) 2001-2006.

(b) This is sample used for all analyses, except as noted.

(c) Percentage point change reflects predicted changes in smoking and drinking pattern response to a $1 increase in state cigarette price.

(d) N = 1,050,573. Includes observations for 2001-2005 only due to change in definition of binge drinking in 2006.

Cigarette price was associated with current, binge, and heavy drinking differentially by age group. For current drinking, a $1 increase in cigarette price was associated with an increase in drinking prevalence of 3.72 percentage points for those aged 65 and older, and a reduction of 4.02 percentage points for those aged 18-20. A $1 increase in price was associated with an increase in binge drinking of 4.06 percentage points for those aged 21-29, and a reduction of 1.95 percentage points for those aged 65 and older. Finally, an increase in cigarette price was associated with a significant increase of 1.15 percentage points in heavy drinking for those aged 21-29, a population that already has higher rates of drinking. An increase in cigarette price did not significantly impact any drinking pattern for those adults 30-64.

## Discussion

This study examined the associations of state cigarette prices with smoking, and current, binge, and heavy drinking by age group. The study’s novel findings are that increases in cigarette price are associated with increased smoking and drinking among those 65 and older, and increased binge and heavy drinking among those aged 21-29. These associations were not necessarily in the directions initially hypothesized. As shown in Table 3, the hypothesis that increases in state cigarette prices would produce stronger reductions in smoking and stronger increases in current, binge, and heavy drinking among young adults (those under 30 years of age), than among those aged 30-64 and 65 and older did not always hold.

In terms of current smoking, the response for those aged 30-64 mirrored the bulk of the literature: an increase in cigarette price was associated with a decrease in smoking [10,11]. However, as indicated in Table 4*,* smoking among the age groups under 30 was not associated with a significant response to cigarette price in this study. This finding is similar to Sloan and Trogdon, who also did not detect significant associations in a study including those aged 21-24 [62]. While a longitudinal study by Tauras, et al. concluded that increases in cigarette price reduced smoking prevalence among youth and young adults, the respondents in that sample were aged 12-22 [49]. Those younger age groups may have responded differently than the categories of 18-29 year olds used in this study. The finding that those aged 65 and older increased smoking prevalence at higher cigarette prices was unexpected, though cigarette price studies of this specific age group are rare [38,63].

For current drinking, and consistent with some other studies [14,64], this study found that higher state cigarette prices were associated with significantly lower rates of current drinking among those aged 18-20, who are not able to purchase alcohol legally for their general consumption. For those aged 65 and older, an increase in cigarette price was associated with a significant increase in rates of current drinking. Another study also found increases in cigarette price associated with increased drinking consumption in older adults [38]. It is possible that increases in cigarette price may be the final motivation for those aged 65 and older to stop smoking, and they may potentially substitute a drink for their smoking. For this age group, which is often on limited budgets, a drink now and then may be less expensive than maintaining smoking [65]. Although this age group has lower overall rates of use, even current drinking may be unhealthy, as its members are more likely than younger age groups to suffer from multiple chronic health conditions, and experience interactions with medications that may be further complicated by drinking. This is a particularly important consideration as the U.S. faces a growing aging population. The response of current drinking to changes in cigarette price of those aged 21-64 was not significantly different from zero.

A concerning finding from this study is that higher cigarette prices were associated with higher rates of both binge and heavy drinking among those aged 21-29, the age group that already has the highest prevalence of smoking and current, binge and heavy drinking of any age group. The response of binge and heavy drinking to cigarette price did not vary across the other age groups, with the exception of a reduction in binge drinking for those aged 65 and older. Only one other study, by Dee [14], compared different forms of drinking outcomes to increases in cigarette taxes among teens. While his models with fewer co-variates found a complementary relationship between cigarette tax and heavy drinking among teens (i.e. as tax increases, drinking decreases), in the more complex models adjusting for state and year effects, Dee also did not find a significant association between cigarette tax and heavy drinking among teens. In the present study, an increase in cigarette price was associated with an increase in current drinking for those aged 65 and older, perhaps due to substituting drinking for smoking. However, it is plausible that smokers in this age group who were binge drinkers might have a different response to increases in cigarette price. It is possible when cigarette prices increased, smokers who both smoked and binge-drank quit both behaviors, but this would not have been detectable in pseudo-panel data. In order to discern these relationships further, longitudinal studies that track the same individuals over time are needed.

Previous studies have found that drinking increases in response to increases in cigarette price [13,18,38], but those studies did not compare responses across age groups. The findings about associations with increased binge and heavy drinking among adults aged 21-29 underscore the importance of examining different patterns of drinking in response to cigarette price across age groups. Future research could: 1) investigate associations interacting age group with other demographic variables (e.g. gender, race/ethnicity) related to smoking and drinking outcomes, and 2) examine factors (e.g. biological, psychosocial, developmental, marketing) contributing to increased drinking behaviors among certain age groups in response to cigarette price. Such research could provide insight for effective interventions with specific populations.

This study has limitations and strengths. First, BRFSS does not collect data on persons who live in institutions, the military, and the homeless. Second, as a telephone survey, the dataset may have bias due to its under-coverage of those individuals who do not have landlines. BRFSS documentation acknowledges that some populations (e.g. those in the rural areas, African Americans in the South, low income populations, and young adults) are less likely to have landlines and the data may not be representative of those populations [66]. However, the weighting adjustments made using the BRFSS weights minimized the impact of such non- or under-coverage [66]. Third, the BRFSS does not track the same individuals over time, nor does it include variables relating to the level of nicotine and alcohol addiction. Thus, it was not possible to analyze differences in demand response due to addiction, or to an individual’s behavior over time. Fourth, in the years of this study, the BRFSS does not include variables that address the number of cigarettes smoked. This information could have provided more understanding on whether individuals reduce the number of cigarettes smoked (rather than quitting) in response to increases in cigarette price. Fifth, the BRFSS relies on self-report for smoking and drinking. While the amount consumed may be underreported, whether someone participates in smoking and/or drinking may be less prone to misreporting [52]. To the extent that underreporting of amount of alcohol consumed occurred, binge and heavy drinking results may be underestimated. While lacking in some areas, the BRFSS was the only dataset that provided publicly available state identifiers, demographics, and information on the health behaviors critical to this study. Sixth, the beer price variable was based on the price of Heineken, which is commonly used in the literature [52], but may introduce potential measurement bias. Finally, while using state fixed effects addresses bias from state characteristics that do not change over time, it does not address all bias, including uncontrolled variables that change over time. Nonetheless, strengths of this study were the controls for state and year fixed effects, the magnitude of smoke-free laws in a state, and state poverty rates. Much of the recent literature stresses the importance of accounting for these factors, which previous studies may have omitted [67,68].

## Conclusions

In summary, age matters, as cigarette prices can be associated with unintended consequences on smoking and drinking behaviors among and across different age groups. Understanding the effects of cigarette taxation policy on different populations is important, as there has been a trend over the last decade for states to increase cigarette taxes, both to advance public health goals, as well as to fill dwindling state coffers. This study adds some cautionary information to decision-makers regarding the attainment of public health goals. Although increasing cigarette taxes has been used widely to prevent and reduce tobacco use, this study suggests there may be unintended consequences for policymakers to consider. Increases in cigarette taxes may result in reductions in: smoking among those aged 30-64, drinking among those aged 18-20, and binge drinking among those aged 65 and older. However, increases in cigarette price through taxation could increase: smoking and drinking among those 65 and older, and binge and heavy drinking among those aged 21-29. Preparing for potential increases in problematic drinking among these age groups could be considered through targeted programming to populations and outreach to practitioners.

Additionally, while there are many researchers and advocates in the alcohol prevention and tobacco control fields, few study both topics. More interdisciplinary behavioral, economic, and health research would benefit our understanding of the prevention, use and co-use of alcohol and nicotine. Advocates and policy-makers could also collaborate and coordinate on policy-making after research has been conducted and plans made for unintended consequences. To our knowledge, a national, purposive, simultaneous campaign to increase alcohol and tobacco prices together has not been conducted, and it is unclear whether such efforts have occurred on the state level. Before creating a campaign to increase simultaneously taxes on alcohol and tobacco products, economic modeling is needed both to arrive at an optimal tax and to understand potential unintended consequences, like the ones found in this study. Tobacco control and alcohol prevention researchers, advocates, practitioners, and policymakers should work together to understand and prepare for unintended consequences of tobacco taxation policy.

## Abbreviations

BRFSS, Behavioral risk factor surveillance system survey; LPM, Linear probability model; SE, Standard error.

## Competing interests

The authors declare that they have no competing interests.

## Authors’ contributions

DM conceptualized the study, conducted the analyses, interpreted data, and drafted the article. DH advised on study design and analyses. DH, PF, SR, and CH contributed to interpretation and manuscript revisions. All authors read and approved the final manuscript.

## References

[B1] Babor T Caetano R Casswell S Edwards G Giesbrecht N Graham K Gruenewald P Hill L Holder H Homel R Alcohol: No ordinary commodity. Research and public policy 2003 Oxford University Press, New York

[B2] Ending the tobacco problem: A blueprint for the nation 2007 National Academies Press, Washington, DC

[B3] Alcohol-attributable deaths report, average for United States http://apps.nccd.cdc.gov/ardi7060577

[B4] Smoking-attributable mortality, years of potential life lost, and productivity losses — United States, 2000-2004 MMWR Morb Mortal Wkly Rep 2008 57 1226 1228 19008791

[B5] Annual smoking-attributable mortality, years of potential life lost, and economic costs — United States, 1995–1999 MMWR Morb Mortal Wkly Rep 2002 51 300 302 12002168

[B6] Improving the quality of health care for mental and substance-use conditions 2006 The National Academies Press, Washington, DC 20669433

[B7] Coate D Grossman M Effects of alcohol prices and legal drinking ages on youth alcohol use J Law Econ 1988 31 145 171 10.1086/467152

[B8] Cook PJ Tauchen G The effect of liquor taxes on heavy drinking Bell J Econ 1982 13 379 390 10.2307/3003461

[B9] Lewit EM Coate D Grossman M The effects of government regulation on teenage smoking J Law Econ 1981 24 545 569 10.1086/466999

[B10] Warner KE Tobacco policy research: Insights and contributions to public health policy 2006 Jossey-Bass, San Francisco, CA

[B11] Chaloupka FJ Warner K Culyer A, Newhouse J The economics of smoking Handbook of health economics 2000 Elsevier Science, NY 1541 1626

[B12] Cook PJ Moore MJ The economics of alcohol abuse and alcohol-control policies Health Aff 2002 21 120 133 10.1377/hlthaff.21.2.120 11900152

[B13] Decker SL Schwartz AE Cigarettes and alcohol: Substitutes or complements NBER Working Paper Series 2000 National Bureau of Economic Research, Cambridge, MA

[B14] Dee TS The complementarity of teen smoking and drinking J Health Econ 1999 18 769 793 10.1016/S0167-6296(99)00018-1 10847934

[B15] Falk DE Yi HY Hiller-Sturmhofel S An epidemiologic analysis of co-occurring alcohol and tobacco use and disorders Alcohol Res Health 2006 29 162 171 17373404 PMC6527037

[B16] Goel RK Morey MJ The interdependence of cigarette and liquor demand South Econ J 1995 62 451 459 10.2307/1060696

[B17] Jones AM A systems approach to the demand for alcohol and tobacco Bull Econ Res 1989 41 85 105 10.1111/j.1467-8586.1989.tb00282.x

[B18] Markowitz S Tauras JA Substance use among adolescent students with consideration of budget constraints Rev Econ Househ 2009 7 423 446 10.1007/s11150-009-9049-6

[B19] Pindyck RS Rubinfeld DL Microeconomics 4th edn 1998 Prentice Hall, Upper Saddle River, NJ

[B20] Davis TJ de Fiebre CM Alcohol’s actions on neuronal nicotinic acetylcholine receptors Alcohol Res Health 2006 29 179 185 17373406 PMC6527039

[B21] Funk D Marinelli PW Le AD Biological processes underlying co-use of alcohol and nicotine: neuronal mechanisms, cross tolerance, and genetic factors Alcohol Res Health 2006 29 186 192 17373407 PMC6527043

[B22] Grucza RA Bierut LJ Co-occurring risk factors for alcohol dependence and habitual smoking Alcohol Res Health 2006 29 172 178 17373405 PMC6527048

[B23] Madden PA Bucholz KK Martin NG Heath AC Smoking and the genetic contribution to alcohol-dependence risk Alcohol Res Health 2000 24 209 214 15986715 PMC6709744

[B24] Little HJ Behavioral mechanisms underlying the link between smoking and drinking Alcohol Res Health 2000 23 215 224 15986716 PMC6709747

[B25] Cigarette smoking among adults—United States, 2007 MMWR Wkly 2008 57 1221 1226 19008790

[B26] Substance A Mental Health Services A Results from the 2006 National Survey on Drug Use and Health: National finding 2007 U.S. Department of Health and Human Services, Substance Abuse and Mental Health Services Administration, Office of Applied Studies, Rockville, MD

[B27] Lucas JW Schiller JS Benson V Summary health statistics for U.S. adults: National Health Interview Survey, 2001 Vital statistics 2004 National Center for Health Statistics, Washington, DC 15791758

[B28] Blazer DG Wu LT Patterns of tobacco use and tobacco-related psychiatric morbidity and substance abuse among middle-aged and older adults in the U.S Aging Ment Health 2012 16 296 304 10.1080/13607863.2011.615739 22292514 PMC3323715

[B29] Lee MR Chassin L Mackinnon D The effect of marriage on young adult heavy drinking and its mediators: Results from two methods of adjusting for selection into marriage Psychol Addict Beh 2010 Dec 24 712 718 10.1037/a0020983 PMC3058715 21198229

[B30] Waldron M Heath A Lynsky M Bucholz K Madden P Martin N Alcoholic marriage: later start, sooner end Alcohol Clin Exp Res 2011 April 35 632 642 10.1111/j.1530-0277.2010.01381.x 21244438 PMC3066284

[B31] Garrett B Dube S Trosclair A Caraballo R Pechacek T Cigarette Smoking — United States, 1965–2008 MMWR 2011 60 109 113 21430635

[B32] National Cancer I The role of media in promoting and reducing tobacco use 2008 U.S. Department of Health and Human Services, National Institutes of Health, National Cancer Institute. NIH Pub. No. 07-6242, Bethesda, MD

[B33] Saffer H Alcohol advertising and alcohol consumption by adolescents Heal Econ 2006 15 617 637 10.1002/hec.1091 16475245

[B34] Bobo JK Husten C Sociocultural influences on smoking and drinking Alcohol Res Health 2000 24 225 232 15986717 PMC6709745

[B35] Gruenewald PJ Ponicki WR Holder HD The relationship of outlet densities to alcohol consumption: a time series cross-sectional analysis Alcohol Clin Exp Res 1993 17 38 47 10.1111/j.1530-0277.1993.tb00723.x 8452207

[B36] Adams S Cotti C Drunk driving after the passage of smoking bans in bars J Public Econ 2008 92 1288 1305 10.1016/j.jpubeco.2008.01.001

[B37] Pindyck Robert S Rubinfeld Daniel L Microeconomics 1998 Fourth Prentice Hall, Upper Saddle River, NJ

[B38] Picone G Sloan F Trogdon J The effect of the tobacco settlement and smoking bans on alcohol consumption Health Econ 2004 13 1063 1080 10.1002/hec.930 15386690

[B39] Bask M Melkersson M Rationally addicted to drinking and smoking? App Econ 2004 36 373 381 10.1080/00036840410001674295

[B40] Cameron L Williams J Cannabis, alcohol, and cigarettes: substitutes or complements? Econ Rec 2001 77 19 34 10.1111/1475-4932.00002

[B41] Substance A Mental Health Services A Results from the 2005 National Survey on Drug Use and Health: National Findings 2006 SAMHSA Office of Applied Studies, Rockville, MD

[B42] Vital signs: current cigarette smoking among adults aged ≥18 years –- United States, 2005–2010 MMWR Wkly 2011 60 1207 1212 21900875

[B43] BRFSS Annual Survey Data http://www.cdc.gov/brfss/technical_infodata/surveydata.htm7060577

[B44] Allison PD Missing data 2002 Sage Publications, Thousand Oaks, CA

[B45] Behavioral risk factor surveillance system survey data 2006 U.S. Department of Health and Human Services, Centers for Disease Control and Prevention, Atlanta, GA

[B46] The tax burden on tobacco, historical compilation Vol 42 2007 Orzechowski and Walker, Arlington, VA

[B47] Tauras J Smoke-free air laws, cigarette prices, and adult cigarette demand Econ Inq 2006 44 333 342 10.1093/ei/cbj028

[B48] Consumer price index ftp://ftp.bls.gov/pub/special.requests/cpi/cpiai.txt7060577

[B49] Tauras J Markowitz S Cawley J Lindgren J Tobacco control policies and youth smoking: Evidence from a new era Substance use: Individual behavior, social interactions, markets and politics Advances in health economics and health services research 2005 Elsevier, New York 277 291 17867244

[B50] Prior HHS Poverty Guidelines and Federal Register References http://aspe.hhs.gov/POVERTY/figures-fed-reg.shtml7060577

[B51] Inter-city cost of living index Inter-city cost of living index 2001-2006 American Chamber of Commerce Researchers Association, Arlington, VA

[B52] Cook PJ Moore MJ Culyar AJ, Newhouse JP Alcohol Handbook of health economics 2000 Elsevier Science, NY 1630 1672

[B53] Ross H Chaloupka FJ The effect of public policies and prices on youth smoking South Econ J 2004 70 796 815 10.2307/4135273

[B54] State smoke-free indoor air fact sheet http://apps.nccd.cdc.gov/statesystem/publications/STATEsystemfactsheetsmokefree.pdf7060577

[B55] Tynan M Babb S MacNeil A State smoking restrictions for private-sector worksites, restaurants, and bars – United States, 2004 and 2007 MMWR Morb Mortal Wkly Rep 2008 57 549 552 18496503

[B56] Wooldridge J Introductory econometrics 2006 3 Thomson South-Western, USA

[B57] Ai C Norton E Interaction terms in logit and probit models Econ Lett 2003 80 123 129 10.1016/S0165-1765(03)00032-6

[B58] Domino M Norton E Morrissey J Thakur N Cost shifting to jails after a change to managed mental health care Health Serv Res 2004 39 1379 1402 10.1111/j.1475-6773.2004.00295.x 15333114 PMC1361075

[B59] Liu Z Dow W Norton E Effect of drive-through delivery laws on postpartum length of stay and hospital charges J Health Econ 2004 23 129 155 10.1016/j.jhealeco.2003.07.005 15154691

[B60] The SURVEYREG procedure 2008 SAS Institute Inc, Cary, NC

[B61] Table 1. Population by Age, Sex, Race and Hispanic Origin: March 2002 Detailed Tables (PPL-167) http://www.census.gov/population/age/data/files/2002/table1.pdf7060577

[B62] Sloan F Trogdon J The impact of the master settlement agreement on cigarette consumption J Policy Anal Manag 2004 23 843 855 10.1002/pam.20050 15499706

[B63] DeCicca P McLeod L Cigarette taxes and older adult smoking: evidence from recent large tax increases J Health Econ 2008 27 918 929 10.1016/j.jhealeco.2007.11.005 18178277

[B64] Goodman AC Economic analysis of multiple addictions for men and women J Ment Health Policy Econ 2009 12 139 156 19996476

[B65] Merrick EL Horgan CM Hodgkin D Garnick DW Houghton SF Panas L Saitz R Blow FC Unhealthy drinking patterns in older adults: prevalence and associated characteristics J Am Geriatr Soc 2008 56 214 223 10.1111/j.1532-5415.2007.01539.x 18086124

[B66] Comparability of data: BRFSS 2006 http://www.cdc.gov/brfss/technical_infodata/surveydata/2006.htm7060577

[B67] Carpenter C Cook PJ Cigarette taxes and youth smoking: new evidence from national, state, and local Youth Risk Behavior Surveys J Health Econ 2008 27 287 299 10.1016/j.jhealeco.2007.05.008 18242745

[B68] Tauras JA Differential impact of state tobacco control policies among race and ethnic groups Addiction 2007 102 Suppl 2 95 103 17850619 10.1111/j.1360-0443.2007.01960.x

